# An educational game for teaching clinical practice guidelines to Internal Medicine residents: development, feasibility and acceptability

**DOI:** 10.1186/1472-6920-8-50

**Published:** 2008-11-18

**Authors:** Elie A Akl, Reem Mustafa, Thomas Slomka, Alia Alawneh, Abhishek Vedavalli, Holger J Schünemann

**Affiliations:** 1 Department of Medicine, State University of New York at Buffalo, NY, USA; 2 Department of Family Medicine, State University of New York at Buffalo, NY, USA; 3 Educational Technology Center, State University of New York at Buffalo, NY, USA; 4 Digital Library Center, State University of New York at Buffalo, NY, USA; 5 CLARITY Research Group, Department of Clinical Epidemiology & Biostatistics, McMaster University, Hamilton, ON, Canada; 6 Department of Epidemiology, Italian National Cancer Institute Regina Elena, Rome, Italy

## Abstract

**Background:**

Adherence to Clinical Practice Guidelines (CPGs) remains suboptimal among internal medicine trainees. Educational games are of growing interest and have the potential to improve adherence to CPGs. The objectives of this study were to develop an educational game to teach CPGs in Internal Medicine residency programs and to evaluate its feasibility and acceptability.

**Methods:**

We developed the Guide-O-Game^© ^in the format of a TV game show with questions based on recommendations of CPGs. The development of the Guide-O-Game^© ^consisted of the creation of a multimedia interactive tool, the development of recommendation-based questions, and the definition of the game's rules. We evaluated its feasibility through pilot testing and its acceptability through a qualitative process.

**Results:**

The multimedia interactive tool uses a Macromedia Flash web application and consists of a manager interface and a user interface. The user interface allows the choice of two game styles. We created so far 16 sets of questions relating to 9 CPGs. The pilot testing proved that the game was feasible. The qualitative evaluation showed that residents considered the game to be acceptable.

**Conclusion:**

We developed an educational game to teach CPGs to Internal Medicine residents that is both feasible and acceptable. Future work should evaluate its impact on educational outcomes.

## Background

Researchers have evaluated a number of strategies to improve the implementation of clinical practice guidelines (CPGs). These strategies include didactic sessions, passive dissemination of information, printed educational materials, audit and feedback, interactive workshops, use of local opinion leaders, and computerized decision support systems. However, the effects of these interventions vary from trivial to moderately large [1]. Research evidence also suggests a higher efficacy of multifaceted interventions and underlines the importance of tailoring interventions to specific barriers and particular settings [1,2].

An educational game is defined as a competitive activity with a prescribed setting constrained by rules and procedures [3]. Learning results from peer interaction and feedback in an entertaining and low risk environment. By allowing active learning experiences, educational games stimulate higher thinking such as analysis, synthesis, and evaluation [4]. They make the learning process fun and exciting and reduce stress and anxiety, which in turn may increase retention [5]. They also can generate useful points of departure for discussion [6]. In a national survey of internal medicine program directors, 90% supported the use of educational games as an educational strategy while 78% reported already using educational games (unpublished data).

Thus, educational games represent an educational strategy of growing interest and has the potential to improve adherence to CPGs [7,8]. Indeed, these games could be used in multifaceted interventions, could be tailored to the particular setting of residency training, and would address two of the major barriers to adherence to CPGs [9]: the lack of awareness of CPGs and the lack of familiarity with their recommendations. Formal and informal discussions with internal medicine residency program directors and chief residents at the national and local level revealed a need for additional strategies for teaching CPGs with an interest in educational games as a potential strategy.

The objectives of this study were to develop an educational game to teach CPGs in Internal Medicine residency programs and to evaluate its feasibility and acceptability.

## Methods

### Developing the game

We developed the Guide-O-Game^© ^following the format of TV game shows in which two teams of residents compete in answering questions that are based on recommendations of CPGs. We initially designed the game for teams instead of single users to compete in order to use it in group educational activities (e.g. noon conference) and allow the maximum number of residents to actively participate. The development of the Guide-O-Game^© ^consisted of: (1) the creation of a multimedia interactive tool, (2) the development of recommendation-based questions, and (3) the definition of the rules of the game.

A team of instructional technology specialists from the Educational Technology Center of the University at Buffalo  programmed the multimedia interactive tool. We followed a systematic approach for developing the recommendation-based questions of the Guide-O-Game^© ^(Additional file 1). The approach included 3 steps: (1) developing a comprehensive list of guidelines for potential inclusion; (2) assessing these guidelines for inclusion, including a methodological quality assessment using the Appraisal of Guidelines Research and Evaluation (AGREE) instrument [10]; (3) developing questions based on the recommendations of the included guidelines. We tailored the Guide-O-Game^© ^in order to the specific context of residents and clinical practice guidelines and healthcare issues. Indeed, the pilot testing (see below) confirmed the need for such tailoring.

### Evaluating feasibility

We pilot tested the Guide-O-Game^© ^in order to evaluate its feasibility in terms of integration in the curriculum, the functionality of its tool, the structure of its questions, and the usability of its rules. We conducted four weekly sessions of the game at a hospital training site for the Internal Medicine residency program at the University at Buffalo. A senior resident (RM) doing an EBM elective run the sessions [11] and participants consisted of a convenience sample of 30 residents rotating at the training site at that time. Each session lasted 45 minutes and was followed by 15 minutes qualitative feedback sessions. The feedback consisted of answering in writing open ended questions followed by an open verbal group discussion. We improved each of the three components of the game (tool, questions, and rules) through an iterative process of pilot testing, feedback and revision.

### Evaluating acceptability

The 15 minutes qualitative feedback sessions included questions about the acceptability of the game in terms of interest in the educational strategy and engagement in the learning process. We used the results of the evaluation after each session to improve the game prior the following session. The Health Sciences Institutional Review Board at the University at Buffalo approved the study.

## Results

### The game

**Figure 1:**
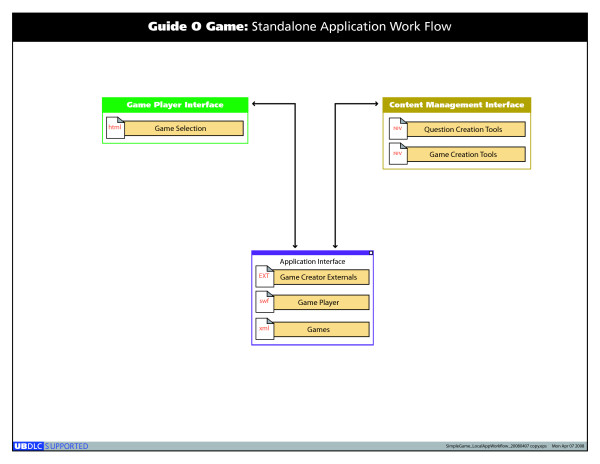
Guide-O-Game^© ^question editor.

**Figure 2:**
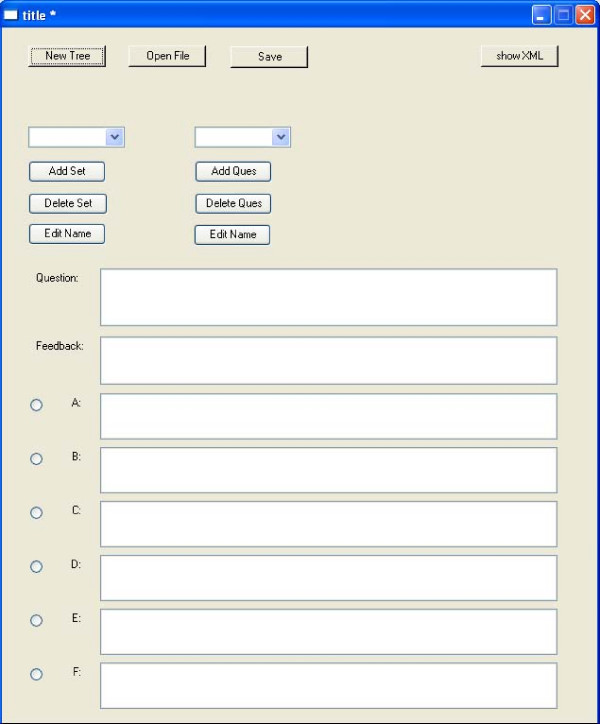
Guide-O-Game^© ^workflow diagram.

Figure 1 shows the tool workflow diagram. The tool uses a Macromedia Flash web application and consists of a manager interface and a user interface compiled by Revolution for Mac and PC. The manager interface allows the creation and editing of questions using a question editor (Figure 2). It also collects data and produces reports about which questions participants selected and what answers they chose. The user interface allows choosing the game settings and running the game. The instructions for the manager interface are available as a users' guide PDF file.

**Figure 3:**
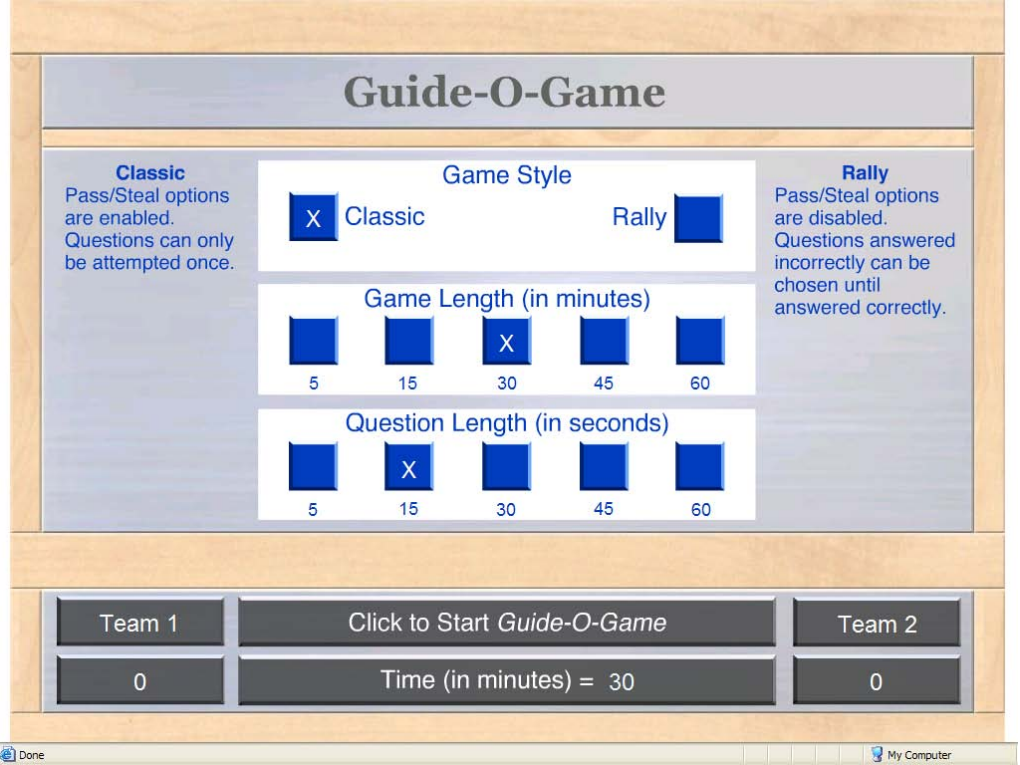
The Guide-O-Game^© ^settings screen.

The user interface settings include the choice of two game styles with different sets of rules (see below), the game length and the question length (Figure 3). Additional features include a soundtrack, the ability to choose the competing teams' names, a countdown clock for the game time, a countdown ticking clock and a timeout sound alert for each question, sounds to indicate whether an answer was correct or incorrect, and automatically calculated and displayed scores. The instructions for the user interface are imbedded the interface itself in order to make it user friendly.

**Figure 4:**
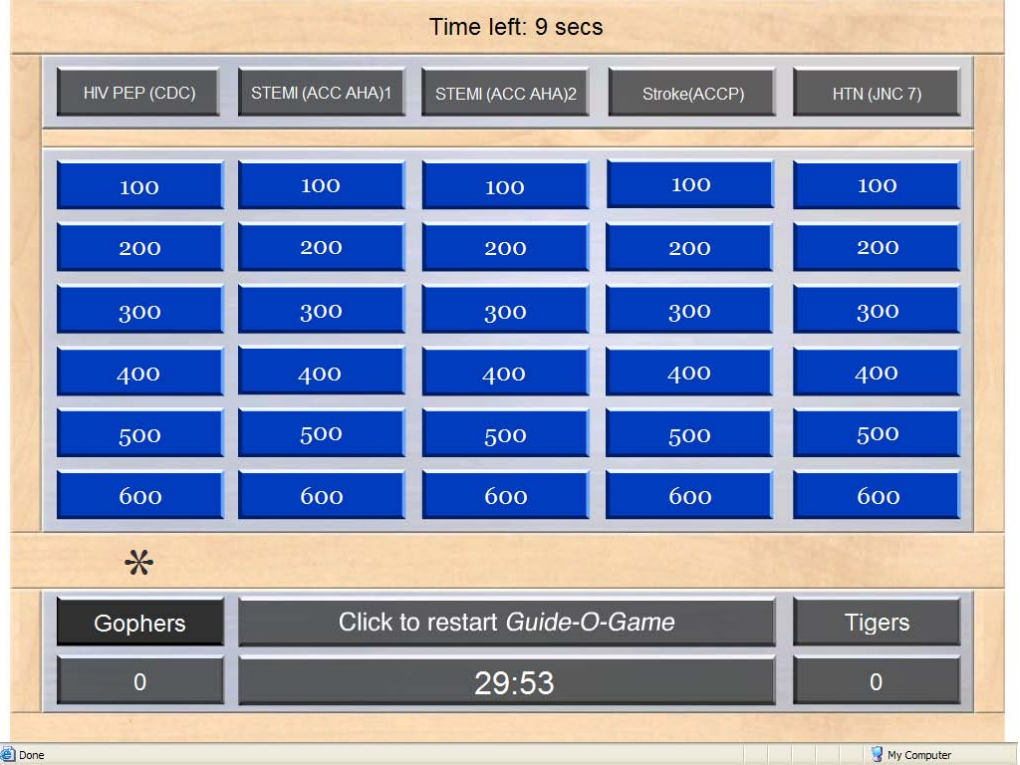
The Guide-O-Game^© ^main screen.

**Figure 5:**
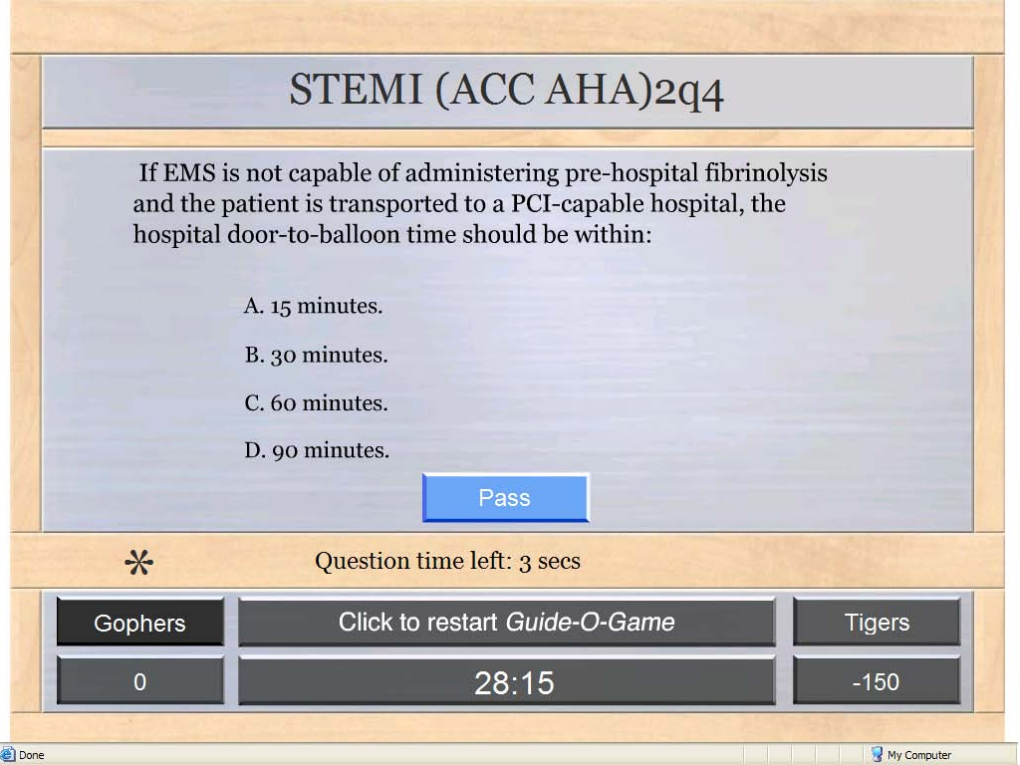
The Guide-O-Game^© ^question screen.

**Figure 6:**
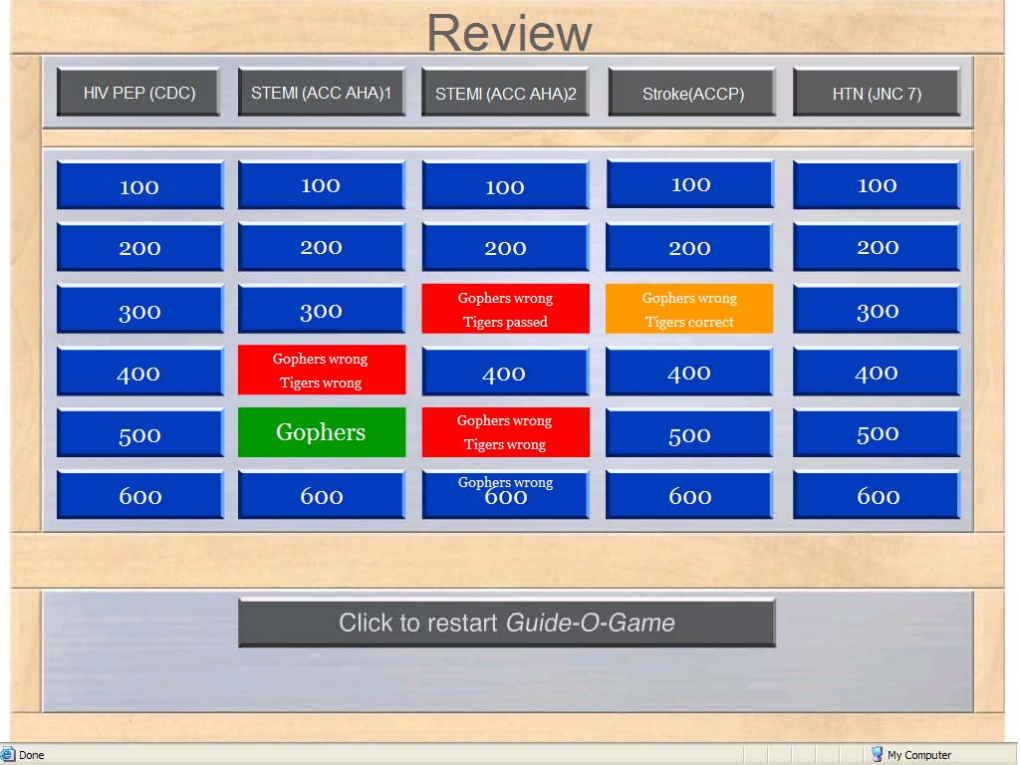
The Guide-O-Game^© ^review screen.

A moderator runs the game on a computer and projects it on a wide screen for participants viewing. The "main screen" displays 5 columns corresponding to 5 different guidelines (Figure 4). Each column has 6 rows corresponding to 6 different questions. The "question screen" presents the question with a number of answer options (Figure 5). After the correct answer is provided, a "rationale screen" provides an explanation for the recommendation (e.g., supporting evidence). The "review screen" is available at the end of the game and shows whether a question was answered correctly or incorrectly and by which team (Figure 6). It thus gives the moderator the opportunity to review questions and to provide the correct answers and the rationale.

We have created 16 sets of "Guide-O-Game^©^" questions relating to nine CPGs (Additional file 2). The user interface allows choosing from two game styles "Classic" and "Rally" that differ by their rules (Additional file 3). The two styles differ mainly by the timing of the provision of the correct answer to an incorrectly answered question. In the classic strategy, designed for learning purposes, the answer is provided before moving to the next question. In the rally strategy, designed for competition purposes, the answer is provided at the end of the game.

Educators can integrate the Guide-O-Game^© ^into the curriculum in different ways. One way would be to include one Guide-O-Game^© ^session per week, with each session lasting 50 minutes followed by a 10 minutes review period. The chief medical resident could serve as the session facilitator and all residents completing their ward rotations and all medical students completing their medicine clerkship would participate. Prizes could be established for the winning team. The residents would be made aware of what guidelines are being "played" the following week. Copies of the guidelines would be made available to interested residents, e.g. through the chief medical resident, rather than distributed to all residents. This is following the first principle of the adult learning theory stating that adults are autonomous and self-directed [12]. We are hypothesizing that residents, as a result of the educational game, would get engaged and take charge of their own learning by getting copies of the guidelines, reading them and becoming familiar with them for the next game session.

### Feasibility

Integrating the four weekly sessions within the curriculum was feasible, although it required ahead-of-time planning. We encountered no major problems with the Guide-O-Game^© ^tool. One glitch with the tool interrupted the first session, but we were able to fix it and the subsequent sessions run smoothly. None of the participants considered that the structure of the questions or their answer options to be confusing. In fact, all discussions around the questions related to their content. We only tested the "Classic Guide-O-Game rules". Because of the slower pace of these rules, we were not able during the assigned 45 minutes to ask all the session questions. Having the full 60 minutes should resolve this issue.

### Acceptability

Participants found the game to be an acceptable educational strategy. First, the game format raised the residents' interest in guidelines recommendations which they described as "a dry material". The interest further increased when we integrated a rationale for each recommendation in the game. Second, they felt playing the game is fun, especially when racing against time to select an answer and when the scores of the two teams were close. This helped them "relax a bit" during a time of the day when work pressure is high (sessions were run during noon conference time). Third, as a result of the fun and the increased interest, the residents reported being more engaged in the learning process in comparison with usual didactic lecture during which they become "easily distracted". Fourth, while residents became actively engaged during the session there were some indicators that they might have also become actively engaged after the session. Indeed, one resident asked which CPGs were to be played the following week so she could "review them in advance".

While participants found most of the questions relevant to their practice, they judged some of them as non-relevant. One example relates to the number of procedures a laboratory need to perform per year to be considered an appropriate percutaneous coronary intervention center. They also criticized not providing a rationale for each recommendation in the initial version of the tool. We did subsequently add such rationale which proved helpful in increasing residents' interest. The residents finally recommended having a review period at the end of each session which we used for discussing some of the answers and rationales.

## Discussion

We have developed an educational game to teach CPGs in Internal Medicine residency programs. It has the format of TV game show using a multimedia interactive tool and allows two teams of residents to compete in answering questions based on recommendations of CPGs. Integrating the game in the curriculum is both feasible and acceptable to residents, in particular because educational games are judged acceptable by the directors of Internal Medicine residency programs in the US. In fact, 78% reported already using educational games (unpublished data).

The Guide-O-Game^© ^has a number of shortcomings. First, it requires frequent updates as new guidelines or updates of guidelines are published. Second, because only two teams compete, there might be too many participants per team which could negatively affect the learning experience of some of these participants. Third, the Guide-O-Game^© ^addresses only 2 of the 7 general categories of barriers to physicians' adherence to CPG (i.e., lack of awareness and lack of familiarity). Consequently it would be ideal to implement it as part of a multifaceted interventions [1]. Finally, educational games in general can be expensive to implement, and time consuming to develop [13]. The evaluation of the acceptability of the game was limited by our use of a convenience sample of residents. Also because of the qualitative nature of the evaluation, we used a non-standardized non-validated measurement tool.

The Guide-O-Game^© ^has a number of strengths. First, it requires less faculty preparation than other educational interventions because it could be made available with ready-to-use questions. This is advantageous for teaching evidence based medicine in general [14], and particularly because time constraints on faculty is reportedly the major barriers to teaching CPGs in Internal Medicine residency programs (unpublished data). Second, the tool can be used by 2 competing single users (instead of teams) or as a tutorial for individual review of CPGs questions. Third, the Guide-O-Game^© ^tool can be adapted for teaching in other specialty residency programs, in countries other than the United States and other content than CPGs. However, this type of educational game is probably most effective for teaching factual information. Indeed, when we initially got interested in exploring the value of educational games in residency training, we focused on guidelines that intended to make clear-cut recommendations and selected strong recommendations based on a clear balance of benefits-downsides.

Other relatively simpler tools, such as Microsoft PowerPoint slide sets with hyperlinks from questions to answer slides, are available on the Internet for adaptation for educational games. The developed interface is superior to those simpler tools for managing the game as it simplifies question creation and editing, allows automated data collection, and produces usage reports. It is also superior for running the game considering the automated scoring, the entertaining sounds, and the imbedded countdown clock.

We are currently developing a web-enabled version of the Guide-O-Game^©^. From the user point of view, the web-enabled version would allow players of different geographical locations to play together (e.g., in a national competition). From the manager point of view, the web-enabled version would allow question developers to upload questions on the Internet and Guide-O-Game^© ^users to download them in a timely and efficient manner. It would also allow centralized collection of performance data (e.g., answers to question) for multicenter research purposes and for validating questions.

A recent systematic review did not identify good quality evidence to confirm or refute the utility of games as a teaching strategy for health professionals [7]. The Guide-O-Game^© ^may potentially improve residents' knowledge of guidelines' recommendations during the educational session through exposure to information during the game sessions. Theoretically, the competitive nature of the Guide-O-Game^© ^may also encourage residents to learn the guidelines' recommendations ahead of the following session in order to be able to win the competition. However, only a trial of high methodological quality can demonstrate the true effect of the Guide-O-Game^© ^on residents' knowledge and clinical behavior [15].

## Conclusion

Using educational games for teaching CPGs is both feasible and acceptable. Medical educators should plan their use in the context of a structured curriculum [16] to address specific educational needs [17]. As an increasing number of similar games are being developed, there is a need for rigorous evaluation of their effectiveness in improving educational and clinical outcomes.

## Competing interests

Elie Akl and Holger Schünemann are respectively participant and co-chair of the eighth American College of Chest Physicians (ACCP) Consensus Conference on Antithrombotic Therapy.

## Authors' contributions

EAA and HJS conceived and designed the study, and obtained funding, TS developed the multimedia tool, EAA, RM, AA, AV and HJS developed the content, EAA drafted the manuscript, and all authors critically revised the manuscript for important intellectual content.

## Pre-publication history

The pre-publication history for this paper can be accessed here:



## Supplementary Material

Additional file 1 **Systematic approach for developing the content of the Guide-O-Game**^**©**^. Click here for file

Additional file 2 **Clinical practice guidelines currently included in the Guide-O-Game**^**©**^. Click here for file

Additional file 3 **Rules of the "Classic" and "Rally" playing strategies.** Click here for file
