# Supplementary material for: An educational game for teaching clinical practice guidelines to Internal Medicine residents: development, feasibility and acceptability
Source: BMC Med Educ. 2008 Nov 18;8:50. doi: 10.1186/1472-6920-8-50 (PMC2631007; doi:10.1186/1472-6920-8-50)
Supplement: Additional file 1 — Systematic approach for developing the content of the Guide-O-Game©. [file 1472-6920-8-50-S1.doc]

Systematic approach for including and developing questions for guidelines for the Guide-O-Game

**Develop a comprehensive list of guidelines for potential inclusion:**

- Ask a number of practicing and in training internists to nominate specific guidelines
- Ask a number of internists to nominate medical professional organizations of relevance to Internal Medicine
- Consider guidelines published by the nominated medical professional organizations and by major organizations (e.g. ACP, ACCP, ATS, ACC, AHA, ADA, etc.)
- Aim to cover the top 6 primary content areas for Internal Medicine as defined by the ABIM: cardiovascular disease, gastroenterology, pulmonary disease, infectious disease, rheumatology/orthopedics, and endocrinology/metabolism (<https://www.abim.org/resources/eiblue_cert.shtm>)

**Assess the guidelines for inclusion**

Inclusion criteria include:

- Relevance of the topic to internal medicine as judged by practicing and in training internists.
- Relevance of the guideline to the US population
- Quality of the guideline assessed using the Appraisal of Guidelines Research & Evaluation Agee (AGREE) instrument ([http://www.agreecollaboration.org/instrument](http://www.agreecollaboration.org/instrument/))
- Date of last update; within 3 years
- Listing on [www.guidelines.gov](http://www.guidelines.gov/)

We aimed to avoid controversial topics

**Develop the questions**

- Follow guidelines derived from the medical literature on how to write multiple choice questions
- Development of questions by one investigator and review by another
- Pilot test questions
