# Supplementary material for: An educational game for teaching clinical practice guidelines to Internal Medicine residents: development, feasibility and acceptability
Source: BMC Med Educ. 2008 Nov 18;8:50. doi: 10.1186/1472-6920-8-50 (PMC2631007; doi:10.1186/1472-6920-8-50)
Supplement: Additional file 2 — Clinical practice guidelines currently included in the Guide-O-Game©. [file 1472-6920-8-50-S2.doc]

Clinical practice guidelines currently included in the Guide-O-Game

| Guideline | Organization | Year of publication | AGREE * score |
| --- | --- | --- | --- |
| Deep venous thrombosis prevention [1] | American College of Chest Physicians (ACCP) | 2004 | 67 |
| Deep venous thrombosis treatment [2] | American College of Chest Physicians (ACCP) | 2004 | 67 |
| Stroke [3] | American College of Chest Physicians (ACCP) | 2004 | 67 |
| Hypertension [4] | Joint National Committee (JNC) 7 report | 2003 | 66 |
| Unstable Angina/Non–ST-Elevation Myocardial Infarction [5] | American College of Cardiology (ACC) and American Heart Association (AHA) | 2007 | 75 |
| ST-elevation myocardial infarction [6] | American College of Cardiology (ACC) and American Heart Association (AHA) | 2007 focused update of the 2004 version | 75 |
| Congestive Heart failure [7] | American College of Cardiology (ACC) and American Heart Association (AHA) | 2005 | 75 |
| Community acquired pneumonia [8] | Infectious Diseases Society of America (IDSA) /American Thoracic Society (ATS) | 2007 | 66 |
| Sepsis [9] | Surviving Sepsis Campaign | 2008 | 71 |

* The AGREE collaboration ([**www.agreetrust.org**](http://www.agreetrust.org/)) recommends that each guideline is assessed by at least two appraisers. The collaboration did not set thresholds for the scores to mark a "good" or "bad" guideline. Guidelines excluded from Guide-O-Game and assessed by AGREE all had scores below 60.

References

[1] Geerts WH, Pineo GF, Heit JA, Bergqvist D, Lassen MR, Colwell CW, et al. Prevention of Venous Thromboembolism: The Seventh ACCP Conference on Antithrombotic and Thrombolytic Therapy. 2004:338S-400.

[2] Buller HR, Agnelli G, Hull RD, Hyers TM, Prins MH, Raskob GE. Antithrombotic Therapy for Venous Thromboembolic Disease: The Seventh ACCP Conference on Antithrombotic and Thrombolytic Therapy. 2004:401S-28.

[3] Albers GW, Amarenco P, Easton JD, Sacco RL, Teal P. Antithrombotic and Thrombolytic Therapy for Ischemic Stroke: The Seventh ACCP Conference on Antithrombotic and Thrombolytic Therapy. 2004:483S-512.

[4] Chobanian AV, Bakris GL, Black HR, Cushman WC, Green LA, Izzo JL, Jr., et al. The Seventh Report of the Joint National Committee on Prevention, Detection, Evaluation, and Treatment of High Blood Pressure: The JNC 7 Report. 2003:289.19.2560.

[5] ACC/AHA 2007 Guidelines for the Management of Patients With Unstable Angina/Non-ST-Elevation Myocardial Infarction: Executive Summary: A Report of the American College of Cardiology/American Heart Association Task Force on Practice Guidelines (Writing Committee to Revise the 2002 Guidelines for the Management of Patients With Unstable Angina/Non-ST-Elevation Myocardial Infarction): Developed in Collaboration with the American College of Emergency Physicians, the Society for Cardiovascular Angiography and Interventions, and the Society of Thoracic Surgeons: Endorsed by the American Association of Cardiovascular and Pulmonary Rehabilitation and the Society for Academic Emergency Medicine. 2007:803-77.

[6] Antman EM, Hand M, Armstrong PW, Bates ER, Green LA, Halasyamani LK, et al. 2007 Focused Update of the ACC/AHA 2004 Guidelines for the Management of Patients With ST-Elevation Myocardial Infarction: A Report of the American College of Cardiology/American Heart Association Task Force on Practice Guidelines: Developed in Collaboration With the Canadian Cardiovascular Society Endorsed by the American Academy of Family Physicians: 2007 Writing Group to Review New Evidence and Update the ACC/AHA 2004 Guidelines for the Management of Patients With ST-Elevation Myocardial Infarction, Writing on Behalf of the 2004 Writing Committee. 2008:296-329.

[7] Hunt SA, Abraham WT, Chin MH, Feldman AM, Francis GS, Ganiats TG, et al. ACC/AHA 2005 Guideline Update for the Diagnosis and Management of Chronic Heart Failure in the Adult: A Report of the American College of Cardiology/American Heart Association Task Force on Practice Guidelines (Writing Committee to Update the 2001 Guidelines for the Evaluation and Management of Heart Failure): Developed in Collaboration With the American College of Chest Physicians and the International Society for Heart and Lung Transplantation: Endorsed by the Heart Rhythm Society. 2005:e154-235.

[8] Mandell LA, Wunderink RG, Anzueto A, Bartlett JG, Campbell GD, Dean NC, et al. Infectious Diseases Society of America/American Thoracic Society Consensus Guidelines on the Management of Community Acquired Pneumonia in Adults. Clinical Infectious Diseases. 2007;44(s2):S27-S72.

[9] Dellinger R, Levy M, Carlet J, Bion J, Parker M, Jaeschke R, et al. Surviving Sepsis Campaign: International guidelines for management of severe sepsis and septic shock: 2008. Intensive Care Medicine. 2008;34(1):17-60.
