# Supplementary material for: An educational game for teaching clinical practice guidelines to Internal Medicine residents: development, feasibility and acceptability
Source: BMC Med Educ. 2008 Nov 18;8:50. doi: 10.1186/1472-6920-8-50 (PMC2631007; doi:10.1186/1472-6920-8-50)
Supplement: Additional file 3 — Rules of the "Classic" and "Rally" playing strategies. [file 1472-6920-8-50-S3.doc]

Rules of the “Classic” and “Rally” playing strategies

**Rally Guide-O-Game**

- Designed for learning purposes
- Two teams compete
- Each team chooses a spokesperson.
- A team is chosen at random to start leading the game.
- The leading team selects a question
- The leading team has ***x*** seconds to provide an answer.
- The team cannot “pass” the question
- If answer is correct: score is increased by the assigned number of points and the team keeps the lead.
- If answer is incorrect or time runs out: score is decreased by half the assigned number of points, the team loses the lead, and the question still be available for competition (on the main screen)
- The other team now leads the game and can choose any question that is available on the main screen (essentially any question not answered correctly yet)

**Classic Guide-O-Game rules**

- Designed for competition purposes
- Two teams compete
- Each team chooses a spokesperson.
- A team is chosen at random to start leading the game.
- The leading team selects a question
- The leading team has x seconds to provide an answer.
- The team cannot “pass” the question
- If answer is correct: score is increased by the assigned number of points and the team keeps the lead.
- If answer is incorrect or time runs out: team loses half the assigned number of points and loses the lead.
- The other team now leads the game and can choose (or not) to answer the last question answered incorrectly by the other team
- If answer is correct: team gains half the assigned number of points.
- If answer is incorrect or time runs out: team loses half the assigned number of points.
